# Genetic association between a single nucleotide polymorphism in Interleukin-16 (rs4072111) and susceptibility to chronic HCV infection in an Iranian population 

**Published:** 2018

**Authors:** Maryam Karkhane, Seyed Reza Mohebbi, Pedram Azimzadeh, Hasti Avarandeh, Shabnam Kazemian, Afsaneh Sharifian, Behzad Hatami, Hamid Asadzadeh Aghdaei

**Affiliations:** 1 *Basic and Molecular Epidemiology of Gastrointestinal Disorders Research Center, Research Institute for Gastroenterology and Liver Diseases, Shahid Beheshti University of Medical Sciences, Tehran, Iran*; 2 *Gastroenterology and Liver Diseases Research Center, Research Institute for Gastroenterology and Liver Diseases, Shahid Beheshti University of Medical Sciences, Tehran, Iran*; 3 *Foodborne and waterborne diseases research center, Research institute for gastroenterology and liver diseases, Shahid Beheshti University of Medical Sciences *

**Keywords:** profile

## Abstract

**Aim:**

Our goal was to identify the putative association of rs4072111 variant in IL-16 gene and HCV susceptibility in an Iranian population.

**Background:**

Interleukin 16 (IL-16), a multifunctional cytokine, plays a vital role in modulation of immune system.

**Methods:**

In present case control and cross sectional study, IL-16 gene variant in 300 patients with hepatitis C (HCV) infection and 300 healthy individuals were analyzed. To evaluate this possible association, genomic DNA from venous blood was extracted and genotypes of IL-16 rs4072111 variant were determined by polymerase chain reaction- Fragments Length Polymorphism Technique (PCR-RFLP). Then, rs4072111 C/T genotypes frequency and allelic distribution were evaluated in each group.

**Results:**

The results of genotyping showed 82% CC, 17.3% CT, 0.7% TT in the control group and 78% CC, 20% CT and 2% TT in the case group. The distribution of rs4072111 C allele was 90.7% in controls and 88% in case group respectively.

However, no correlation between IL-16 rs4072111 C/T variants and susceptibility to chronic HCV infection was found in the present study.

**Conclusion:**

We concluded the rs4072111 C/T cannot be considered as a proper biomarker to identify susceptibility to chronic hepatitis C virus infection.

## Introduction

 Despite the advent of direct acting antiviral (DAA) agents, Hepatitis C virus (HCV) infection is still a matter of concern around the world. HCV is an important reason of chronic hepatitis, liver cirrhosis and hepatocellular carcinoma(1). Approximately, 50 % of patients have successful response to previous standard remedy with pegylated interferon (peg-IFN) plus Ribavirin (2, 3). In spite of intensive efforts in HCV antiviral therapy, definite treatment is not achieved which may due to lack of understanding of the viral contamination process and immune response defects in interaction with HCV (4). On the other hand, no effective vaccine has been designed, produced and applied to prevent HCV infection, yet. 

Emerging observations suggest that both immunologic and virologic events in different stages of infection determine the disease outcome. Spontaneous clearance of HCV observed in 15-45% of acute HCV patients and is associated with a widely specific and powerful cellular immune response. In addition, threshold frequency of CTL is essential for HCV clearance (5, 6).

Immunity against HCV is connecting with vigorous CD4+ T cells and IL-16 plays a crucial role in T cell polarization and T cell response regulation and also, it governs trafficking and biological properties of TCD4+. Interleukin 16 (IL-16) is a potent anti-inflammatory cytokine that is secreted by a variety of cell types such as lymphocyte and epithelial cells. This CD4 co-receptor specific ligand is a multi-functional mediator that initially determined as a lymphocyte chemo-attractant cells (LCF). It can also attract activated T CD4+ cells and re-circulate and activate antigen presenting cells such as monocytes, macrophage and dendritic cells. Although, Il-16 can stimulate the expression of different pro inflammatory cytokines such as IL-1β, IL-4, TNF-α, IL-12 and IL-15 (7, 8). In addition, it works in concert with IL-12 and IL-15 to promote CD4+T cell proliferation and also IL-2α induction on CD4 T cell (9, 10). Most studies emphasize anti -inflammatory property of IL-16 (11, 12). IL-16 gene was located on chr15.q26.3 and genetic polymorphisms in DNA sequence of IL-16 gene may cause to cytokine production and /or activity. Therefore, IL-16 polymorphisms were investigated in several diseases and its association with autoimmune diseases; viral hepatitis B, HBV related HCC and a range of cancers, including prostate, gastric and colorectal cancer has been evaluated (7, 10, 13-16). 

 The possible association between SNPs of IL-16 gene and susceptibility to chronic HCV, has not been investigated in Iranian population, therefore we decided to perform this study for the first time to assess the role of rs4072111 T/C polymorphism HCV chronic infection in a group of Iranian HCV patients in comparison with healthy controls. 

## Methods

**Study population**

In this case-control study, 300 patients with chronic HCV were collected as case group and 300 healthy individuals were studied as control group. Gastroenterology and Liver Diseases Research institute, Shahid Beheshti University of Medical Sciences provided the funding and supported HCV patients and healthy controls sample collection process. Healthy individuals with negative results for anti HCV antibody by ELISA and also without a history of liver associated diseases were included in the study. Selection criteria for patients group defined as negative results for HBs antigen, HBc antibody (Ab) and positive for anti-HCV Ab via enzyme linked immunosorbent assay (ELISA) tests (Diapro Diagnostics, Italy). Patients with Hepatitis B virus co-infection were excluded from the study. 

**DNA extraction**

Genomic DNA was extracted from peripheral blood cells of patients and healthy control subjects by using standard phenol-chloroform extraction method (17). The appropriate quality and standard quantity of the extracted DNA were then checked by NanoDrop Spectrophotometer (Thermo Fisher Scientific Inc; Waltham, MA). 

**IL-16 genotyping**

In order to determine genotypes of IL-16 rs4072111, Restriction fragments length polymorphism (PCR-RFLP) method was utilized following the polymerase chain reaction (PCR) amplification.

The PCR conditions for amplification of target sequence were as follows: 95°C for 5 min; 35 cycles of 95°C for 30 s, 58.4°C for 30 s, and 72°C for 30 s; 72°C for 10 min as last extension. The sequence of primers included forward primer: 5′- CACTGTGATCCCGGTCCAGTC-3′ and reverse primer: 5′- TTCAGGTACAAACCCAGCCAG C -3′ (18) that amplified PCR product about 164bp. 

The final volume of PCR reaction was 25 µl which comprised of 100 ng of template, 200 µM dNTPs (Fermentas, Latvia), 10 pmol of forward and reverse primers, 1.25 U of Taq polymerase.

The enzymatic digestion process for differentiation of rs4072111 genotypes was performed by BsmAI restriction enzyme (Thermoscientific, USA) at 37˚C overnight.

The final products of digestions were run on 2.5% w/v agarose (Hoffmann la Roche AG, Basel, Switzerland) gel, which were stained with green viewer to visualize the fragments patterns by using UV documentation instrument (Vilber Lourmat, France). 

**Statistical analysis**

The demographic data of chronic HCV patients and healthy subjects were shown as frequency (percentage) and mean ± standard error of mean. T-test and Chi-square tests were used to evaluate the data. The adjusted logistic regression analysis was used to survey the possible association between rs4072111 C/T variants or allelic frequencies and susceptibility to chronic HCV infection. To assess this survey odd ratio (OR) and confidence interval 95% (95% CI) illustrated the results. Statistical analysis was two sided, and p<0.05 was considered significant. 

## Results

A total of 600 subjects were included in this study, containing 300 healthy controls and 300 chronic HCV patients. Control group contained 152 (50.7%) males and 148 (49.3%) females and case group comprised of 236 (78.7%) male and 64 female (21.3%) subjects. Along with the random sampling, we tried to select both genders equally in control group, in versus of the case group, which male gender was dominant. 

The mean age of control and case groups was 45.13 ± 0.95 and 44.88 ±0.82 years respectively. Although the mean age of control group was slightly higher than case group, the difference was not significant (p=0.84). 

We studied the distribution of rs4072111 C/T variants in healthy subjects and chronic HCV patients in an Iranian population that the details were illustrated in table 1. The rs4072111 CC was the most frequent variant in both healthy control and patient groups. However, no meaningful difference was observed in the distribution rs4072111 C/T variants in case and control groups.

**Table 1:** The distribution pattern of rs4072111 C/T variants and associated allelic frequency

SNP Variants	Control Group (n=300)	Case Group (n=300)	Adjusted a OR (95% CI), P value
CC	246 (82%)	234 (78%)	1.00,Reference group
CT	52 (17.3%)	60 (20%)	1.18 (0.771-1.825); 0.437
TT	2 (0.7%)	6 (2%)	2.98 (0.563-15.844); 0.199
Alleles			
C	544 (90.7%)	528 (88%)	1.00, Reference group
T	56 (9.3%)	72 (12%)	1.33 (0.918-1.934); 0.132

**Table 2:** Separation of rs4072111 C/T variants and related allelic distribution by gender

Male gender			Female gender			
SNP Frequency						
	Control group	Case group	Control group		Case group	
CC	127 (83.6%)	180 (76.3%)	119 (80.4%)		54 (84.4%)	
CT	24 (15.8%)	51 (21.6%)	28 (18.9%)		9 (14.1%)	
TT	1 (0.7%)	5 (2.1%)	1 (0.7%)		1 (1.6%)	
Allelic Frequency						
C	340 (87.6%)	330 (85.1%)		204 (96.2%)		198 (93.4%)
T	48 (12.4%)	58 (14.9%)		8 (3.8%)		14 (6.6%)

**Figure 1:**
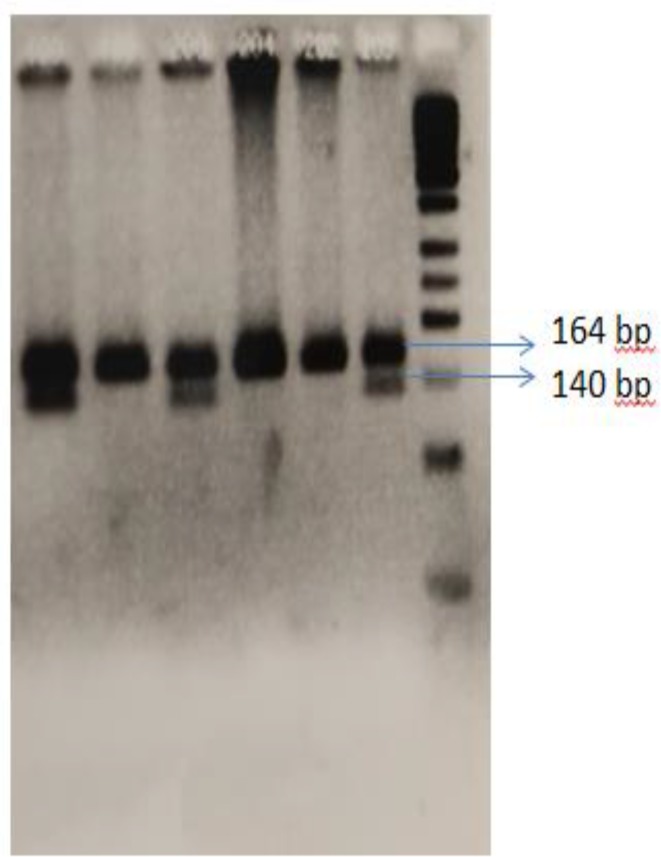
RFLP pattern of IL16 rs4072111 C/T. rs4072111 C and T showed 164 bp and 140 bp along with 24 bp respectively. 50 bp DNA ladder (Thermo Fisher Scientific, USA) was used to distinguish the fragments on the electrophoresis gel

The ancestral allele for position of rs4072111 C/T is C allele. Data analysis of allelic count showed in consistent of high frequency of rs4072111 CC variant, C allele was more frequent rather than T allele in Iranian population.

We also studied the rs4072111 C/T polymorphisms and allelic frequencies in different genders. We observed no significant difference between control and case groups regarding genders. The results were illustrated in table 2. In addition, restriction pattern of PCR product with 164 bp size was depicted in figure 1.

## Discussion

IL-16 is an immonumodulator cytokine which mainly produced by T CD8+. However, macrophage and B cells secrete IL-16 against viral and bacterial infections.

IL-16 plays as chemo-attractant role for certain immune cells expressing the CD4 and assumed as a natural ligand for CD4 molecule which leads to initiation of intracellular pathway through activation of stress-activated protein kinase (SAPK) signaling in T helper CD4+ (19). Lympho-attractant property of IL-16 causes to immune cell recruitment and then, directs the immune response (20). Strong specific T helper CD4+ connected with successful HCV clearance. Therefore, IL-16 with TCD4+ adherence and guidance assume as a major component in immunity against HCV and achievement of spontaneous HCV resolution (21). Moreover, after binding to CD4 molecules, IL-16 triggers the secretion of inflammatory cytokines including TNF-α, IL-1β and IL-4 (22). 

Several researches studied the multifunctional properties of IL-16 in variety range of diseases. In a cell culture based study, Corinne Amiel and colleagues proved a significant inhibitory activity of recombinant IL-16 on viral transcription and replication in human immunodeficiency virus infection (23).

Multiple studies showed anti-inflammatory activity of IL-16 cytokine. Klimiuk PA *et al.* suggest supplemental therapy of rheumatoid arthritis patients with IL-16 as a novel anti-inflammatory treatment (11). In consistent with anti-inflammatory property, Caterina Musolino and colleagues studied the IL-16 concentration in multiple myeloma before treatment and showed high levels of this cytokine in tumor locations (12). 

The patient’s susceptibility associated interaction between immune system and HCV particles well not realized. In addition, several studies have been recently reported association of the most common variants in host genome and susceptibility to viral infections (24-27). The evidences revealed that genetic contents of innate and adaptive immune response may have effect on the natural history of HCV infection (28, 29). Genetic polymorphisms of IL-16 may change the folding of cytokine, biological function and can determine the bio-availability of cytokine. This change in cytokine concentration may cause to alteration of cytokine environment and leads to different outcome of disease (30). 

Several researches showed the role of IL-16 gene polymorphism with auto-immune diseases (31-35). Xue- jiang Gu *et al.* revealed the correlation between Grave’s disease susceptibility and IL-16 gene’s variation (32). In another similar study, variation of IL-16 gene recognized as biomarker for facility of Grave’s autoimmune disease diagnosis (36). Also, -295 T>C polymorphisms in promoter region of IL-16 is associated with increased IL-16 expression which was studied in Crohn’s disease (31). Lew BL *et al.* emphasized the involvement of IL-16 polymorphisms in autoimmune diseases promotion. So that, they showed the rs17875491 and rs11073001 participation in Alopecia areata autoimmune non-scarring hair loss emerging (37).

In the other hand, many issues addressed the major role of IL-16 polymorphism in risk of various kinds of cancers (38-40). Rs4072111 C/T polymorphism was assumed such as risk marker for nasopharyngeal carcinoma occurrence (40). Hughes L *et al.* illustrated the prostate cancer diagnosis and screening strategy via genetic polymorphisms of IL-16 which are related to micro-RNA binding site products. In this assay, rs1131445 TT genotype presumed risk factor for earlier time appearance of prostate cancer among African-American ethnicity (41). 

Rs4072111 was considered as suitable susceptibility biomarker for progression of chronic HBV infection by Romani *et al.* They suggest that, rs4072111 T allele increased in chronic HBV patients compared to healthy individuals (42). 

In addition, Eed Em *et al.* found that rs4072111 CT can consider as HBV biomarker and rs4072111 TT polymorphism increase the risk of HBV-associated HCC in Saudi population(43). These results are consistent with Shan Li *et al.* who showed that significant increase of IL-16 rs4072111 TT in Chinese population with HCC (10). 

To our knowledge, no study was investigated the potent role of IL-16 variants in chronic HCV patients’ susceptibility, up to now. In this research, we evaluated the distribution of rs4072111 C/T in patients with chronic HCV patients in comparison with healthy control group. The results of our research showed no significant difference between distribution of rs4072111 C/T polymorphisms and allelic frequency in chronic HCV patients in comparison to healthy people.

It seems that rs4072111 C/T polymorphism cannot be considered as a susceptibility biomarker of chronic HCV infection and other IL-16 single nucleotide polymorphisms should be analyzed in a larger study group to elucidate the role of this cytokine in chronicity of HCV infection.

## Conflict of interests

The authors declare that they have no conflict of interest.
